# Power Output Enhancement of Natural Rubber Based Triboelectric Nanogenerator with Cellulose Nanofibers and Activated Carbon

**DOI:** 10.3390/polym14214495

**Published:** 2022-10-24

**Authors:** Pongsakorn Mekbuntoon, Walailak Kaeochana, Teerayut Prada, Intuorn Appamato, Viyada Harnchana

**Affiliations:** 1 Department of Physics, Khon Kaen University, Khon Kaen 40002, Thailand; 2 Institute of Nanomaterials Research and Innovation for Energy (IN-RIE), Khon Kaen University, Khon Kaen 40002, Thailand

**Keywords:** natural rubber, triboelectric nanogenerator, cellulose nanofibers, sugar cane bagasse, activated carbon

## Abstract

The growing demand for energy and environmental concern are crucial driving forces for the development of green and sustainable energy. The triboelectric nanogenerator (TENG) has emerged as a promising solution for harvesting mechanical energy from the environment. In this research, a natural rubber (NR)-based TENG has been developed with an enhanced power output from the incorporation of cellulose nanofibers (CNF) and activated carbon (AC) nanoparticles. The highest voltage output of 137 V, a current of 12.1 µA, and power density of 2.74 W/m^2^ were achieved from the fabricated NR–CNF–AC TENG. This is attributed to the synergistic effect of the electron-donating properties of cellulose material and the large specific surface area of AC materials. The enhancement of TENG performance paves the way for the application of natural-based materials to convert mechanical energy into electricity, as a clean and sustainable energy source.

## 1. Introduction

The development of clean and sustainable alternative energy is regarded as an essential solution to the global energy crisis. Energy harvesting from the environment is one of the promising solutions which has drawn much interest. Mechanical energy is the most distributed form of energy in our living environment, but it is often neglected and wasted. The triboelectric nanogenerator (TENG) is a new emerging energy technology that converts mechanical energy in the environment into electricity, based on a combination of two physical phenomena, contact electrification and electrostatic induction [1]. Taking advantage of high energy conversion efficiency, simple structure and fabrication, many operational modes and a variety of material choices, TENG has many potential applications in diverse fields including micro/nano power sources, self-powered sensors, air/gas filtration and control interface [2,3].

Biocompatible and environmentally friendly TENGs have been extensively developed in recent years. There are many natural materials that have been employed to fabricate TENG, such as plant leaf [4], silk [5], wood [6], chitin and chitosan [7,8], cellulose [9], and natural rubber [10,11,12]. Natural rubber (NR) is one of the promising natural materials for the fabrication of a green and highly efficient TENG, since it is a natural polymer extracted from the *Hevea brasiliensis* tree [13] and widely used for manufacturing many industrial products which are used in kinetic situations. In addition, NR possesses many appealing aspects, including good flexibility and tensile strength, the feasibility to make composite materials or to chemically functionalize with tunable properties, and a low cost.

NR is tribopositive when tested with PTFE, which is the most tribonegative material [10,11,12]. Modified NR with improved electron-donating properties to increase positive tribopolarity would improve the electrification effect, giving rise to the enhancement of the TENG performance. Nanocellulose or cellulose nanofiber (CNF) is one of the most abundant green materials, which has been increasingly explored in recent years, due to many fascinating properties including good mechanical properties: excellent stiffness and high strength, low density, the ability to modify its surface chemistry, biodegradable properties and biocompatibility [14,15]. Recently, the CNF-based TENG was reported to have a power density of 300 W/m^2^, which was the highest record of the TENG performance of green materials [16]. Its higher position in the triboelectric series of cellulose (more positive tribopolarity) when compared with NR indicates that cellulose has a high ability to donate electrons [17,18]. In this regard, the incorporation of CNF is anticipated to increase the positive tribopolarity of the NR composite.

Apart from material properties, the electrification effect is also controlled by the size of the friction area [19]. Many surface modification techniques have been proposed, to increase the surface area of triboelectric materials, such as surface-patterning [20,21], plasma etching [22], soft lithography [23], and filling with/structure modification of porous or sponge structure [24,25,26]. In the latter case, porous structured materials have been intensively used for boosting the charge generation and accumulation of friction materials, due to their large specific surface area [27,28,29,30]. Activated carbon (AC) is a highly-porous carbonaceous material with a high surface area [31], which is attractive for modifying the surface structure of the NR composite. So far, there are still very few reports on using AC as a triboelectric material for TENG [12,32].

In the present work, NR is filled with CNF to magnify tribopositive polarity and, by taking advantage of the high surface area, porous AC, to improve the electrification effect in order to enhance the energy conversion performance of the TENG. In this research, CNF is extracted from agricultural waste (sugarcane bagasse), and is then added to NR and AC, forming an NR–CNF–AC composite. The effect of filler concentration on the TENG output and the role of each filler material are investigated. The applications of the fabricated NR-based TENG to charge capacitors and to power light emitting diodes (LEDs) are also demonstrated. This work highlights the use of a combination of CNF and AC, which is found to exhibit a synergistic enhancement of the TENG output performance. The findings of this work may lead to the development of high performance and environmentally friendly TENG, which is a potential power source for micro/nano electronic devices.

## 2. Materials and Methods

### 2.1. Materials and Chemicals

Sugarcane bagasse (SB) was obtained from Mitr Phol S ugar Factory, Phu Khiao, Chaiyaphum, Thailand. Sodium hydroxide (NaOH, 97%) was purchased from KEMAUS (Cherrybrook, Australia). Hydrogen peroxide (H_2_O_2_, 35%) was purchased from ANaPURE (Brightchem Sdn. Bhd., Selangor, Malaysia). Hydrochloric acid solution (HCl, 37%) was obtained from RCI Labscan (Bangkok, Thailand). AC powder, NORIT^®^ RX1.5 EXTRA, was purchased from SIGMA-ALDRICH (St. Louis, MO, USA).

### 2.2. Synthesis of CNF

SB was firstly washed with tap water and oven-dried at 60 °C for 48 h. Dried SB was cut into 1–5 cm pieces, which were then ground into powder form using a grinder machine, to obtain 75 µm SB powder. A total of 15.0 g of the SB fine powder was alkaline treated, using 10% NaOH at a solid/liquid ratio of 1:20 g/mL. The alkaline-treating process was conducted at 90 °C for 4 h. The mixtures were then filtered, and washed with DI water until a neutral pH was achieved. The bleaching treatment was then performed by using alkaline peroxide, a mixture of 25% H_2_O_2_ and 2% NaOH solutions (the volume ratio of H_2_O_2_ to NaOH was 4:1), at 60 °C for 3 h. After this, the bleached product was washed with DI water to reach a neutral pH. The filtrated fibers were then hydrolyzed in a 10% HCl at 90 °C for 2 h. After this, the suspension was washed with distilled water to neutral under a homogenization process at 10,000 rpm for 4 h, to obtain the CNF solution.

### 2.3. Preparation of NR–CNF and NR–CNF–AC Composite Film

The commercial NR latex was purchased from the Thai Rubber Latex Group Public Co., Ltd. (Chonburi, Thailand) with a dry rubber content of 61%. 20 mL NR latex was mixed with the prepared CNF solution at 0.8, 1.2, 1.6 and 2.0 wt% for preparing the NR–CNF composite and with AC powder at 0.2, 0.4 and 0.6 wt% for preparing the NR–CNF–AC composites. The mixtures were then magnetically stirred for 20 min to obtain homogeneous suspensions. A total of 1.5 mL of the mixture was cast on a 4 × 4 cm^2^ ITO substrate, to obtain a film thickness of approximately 1 mm, and three samples were prepared for each experimental condition. The specimens were left to dry at room temperature for 1 day and then cured at 60 °C for 6 h. The samples were then ready for the TENG performance test.

### 2.4. Material Characterizations

CNF was characterized by a TEM (TECNAI G2 20, FEI), a Fourier-transform infrared spectroscopy (FTIR TENSOR27), and XRD (PANalytical EMPYREAN). The morphologies of the NR, NR–CNF and NR–CNF–AC composite films and AC powders were studied using a SEM (Helios Nanolab, FEI) SEM.

### 2.5. TENG Output Measurement

The TENG performance of the NR–CNF composites was tested under a single electrode mode with a contact-separation configuration, using a PTFE sheet as a contact triboelectric material. The contact areas of the two surfaces were 4 × 4 cm^2^. The TENG output voltage and current were measured using an oscilloscope (Tektronix DPO2002B) and a digital ammeter (Keithley DMM6500), respectively. The output signals were acquired during the applied impact force of 1 N at 5 Hz frequency.

## 3. Results and Discussion

**Figure 1:**
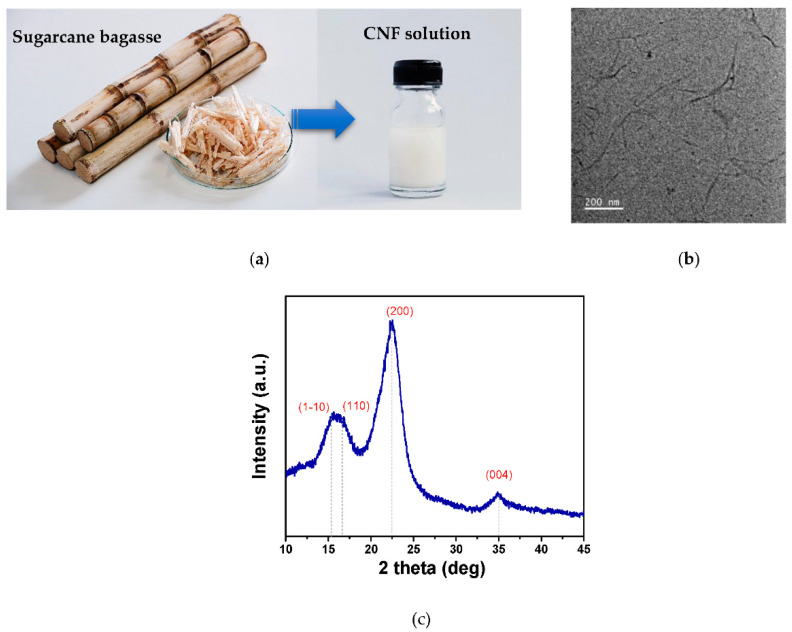
(**a**) CNF solution prepared from sugarcane bagasse used for the fabrication of NR–CNF composite films; (**b**) TEM image of cellulose nanostructure, and (**c**) XRD pattern of the synthesized CNF.

The triboelectric materials were fabricated from the prepared CNF from sugarcane bagasse, as shown in the photograph in Figure 1a. The nanostructure of CNF is confirmed by the TEM image shown in Figure 1b, revealing a fine fiber-like structure with a diameter of a few nanometers and length of a few hundred nanometers. The XRD pattern of the CNF (Figure 1c) reveals the diffraction peaks at *2θ* of 15.3, 16.6, 22.5, and 35.0°, which are indexed to the reflection from (1–10), (110), (200), and (004) planes of cellulose I, respectively [33].

**Figure 2:**
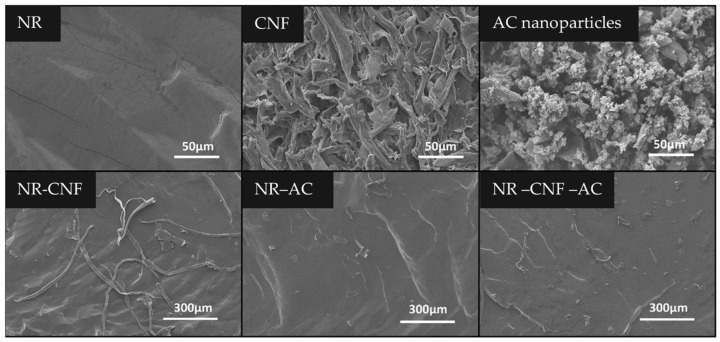
SEM images of NR, CNF, AC powder, and the cross-sections of NR–CNF, NR–AC and NR–CNF–AC composite films.

The morphologies of the NR, CNF, AC powder, and the cross-sections of the NR–CNF, NR–AC and NR–CNF–AC composite films are revealed in SEM images in Figure 2. All the filler particles were clearly visible in the NR composites. Long thin fibers of CNF were detected across the NR–CNF and NR–CNF–AC films, while AC particles with various sizes were observed in the NR–AC and NR–CNF–AC films.

**Figure 3:**
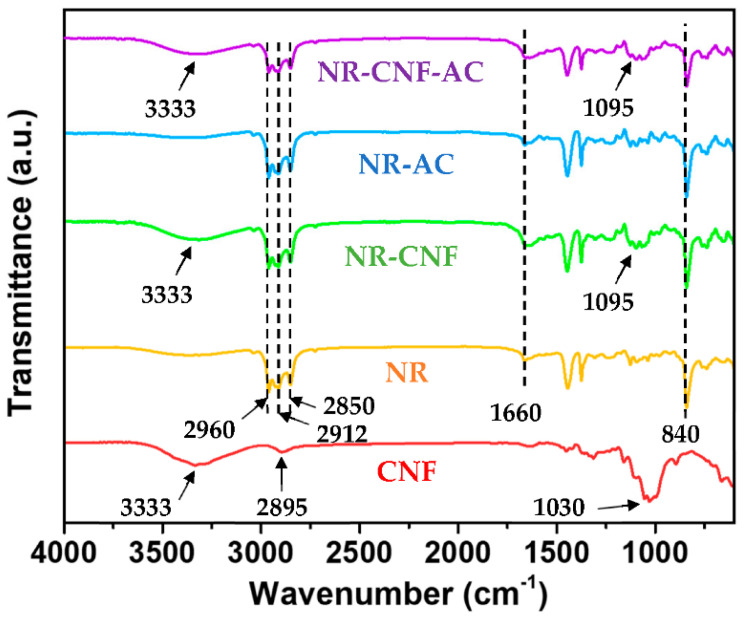
FTIR spectra of CNF, NR, NR–CNF, NR–AC, and NR–CNF–AC composites.

A FTIR analysis was performed, to examine the chemical structure of the fabricated composite films. The FTIR spectra of CNF, pristine NR, NR–CNF, NR–AC and NR–CNF–AC are presented in Figure 3. CNF exhibits main characteristic FTIR peaks located at 1030, 2895, and 3333 cm^−1^, which correspond to a stretching vibration of C–O–C, O–H, and C–H bonds, respectively. Other peaks at 1635 and 895 cm^−1^ are associated with the O-H bending vibration from the adsorbed water on the CNF surface and cellulosic β-glycosidic linkages [34], respectively. The FTIR spectra of pristine NR and all NR composites are similar, and show absorption peaks at 840 and 1660 cm^−1,^ which are assigned to the out-of-plane bending vibration of C–H and C=C stretching of cis-1,4-polyisoprene, respectively [35]. The multiple peaks at 2850–2912 and 2960 cm^−1^ correspond to the asymmetric-symmetric stretching vibration of CH_2_, and C–H in the NR molecule, respectively. In addition, for NR–CNF and NR–CNF–AC composites, the stretching vibration of O–H and C–O–C in CNF are observed at 3333 cm^−1^ and 1095 cm^−1^, respectively, but they are absent in the NR–AC sample. This suggests that the incorporations of CNF and AC do not change the chemical characteristic of NR, since no bonding interaction between CNF and NR is detected. 

**Figure 4:**
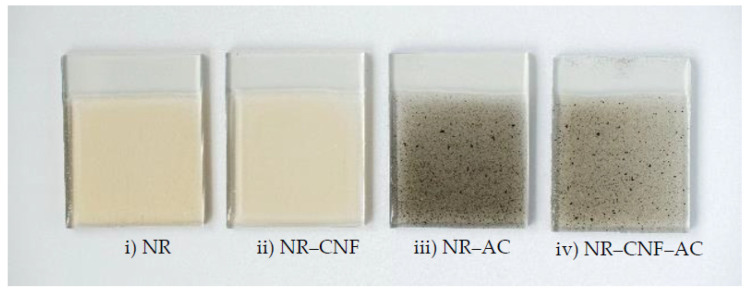
Digital photographs of (**i**) NR, (**ii**) NR–CNF, (**iii**) NR–AC and (**iv**) NR–CNF–AC composite films on ITO substrates.

**Figure 5:**
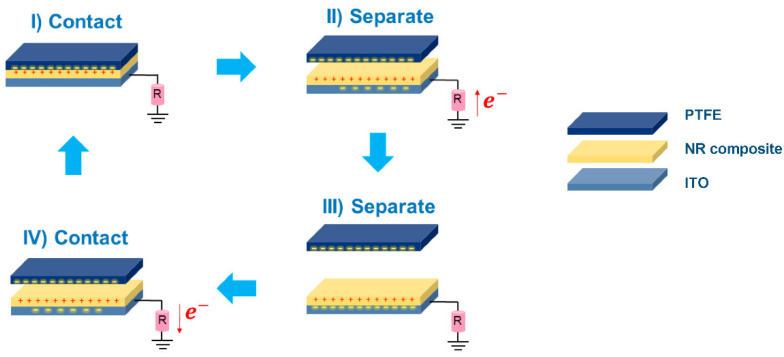
The working mechanism of the NR composite TENG in a single electrode mode.

The photographs of the fabricated NR, NR–CNF, NR–AC (without CNF) and NR–CNF–AC composite films on ITO conductive glasses are presented in Figure 4. They were then used as tribopositive electrodes, and tested with a Teflon sheet as a tribonegative electrode. The generation of electricity of the fabricated TENGs is described in Figure 5. The electrification effect occurs when two surfaces are in contact, which results in the formation of surface charges with different signs. The separation of the two surfaces causes the potential build up that could induce free electrons to flow to neutralize the potential, generating a positive current signal. Once the surfaces are brought back in contact again, the potential is reduced and disappears, and electrons flow back to the ground, generating a negative current.

**Figure 6:**
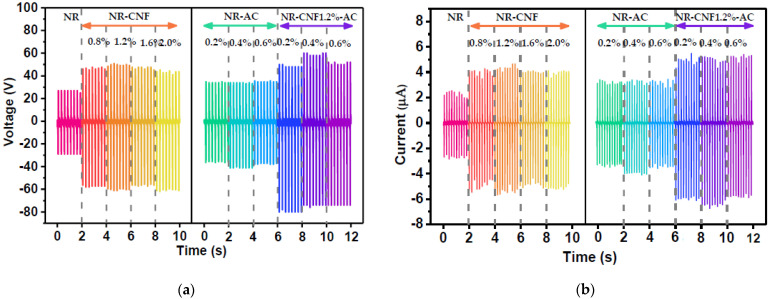
(**a**) Output voltage, and (**b**) current of the fabricated TENGs from NR, NR–CNF, NR–AC and NR–CNF–AC composite films under 1N impact force at working frequency of 5 Hz.

The electrical output voltage and current of the fabricated TENGs are displayed in Figure 6a,b, respectively. The TENG performance was significantly improved with the presence of CNF in the NR films. However, the electrical outputs of the NR–CNF TENGs at a CNF concentration of 0.8–2.0 wt% were not greatly changed. The NR–CNF@1.2% TENG showed the highest peak-to-peak voltage (*V_pp_*), of 113 V, and peak-to-peak current (*I_pp_*), of 10.4 µA. At a higher CNF content, TENG outputs gradually dropped. The enhanced TENG performance was attributed to the positive tribo-polarity of cellulose [17] in the CNF, due to the electron donating ability of oxygen in their hydroxyl functional groups [36,37]. In addition, the enriched hydroxyl function groups help CNF dissolve in NR latex which contains a reasonable amount of water; the higher the CNF fraction, the more liquefied the suspension. In this regard, the thicknesses of the NR–CNF composite films were found to reduce with increasing CNF concentration, and were 0.80, 0.78, 0.75 and 0.72 mm for NR–CNF@0.8%, 1.2%, 1.6% and 2.0%, respectively. Generally, the reduction of triboelectric film thickness (*t*) leads to the enhancement of surface charge density, which is described by the term *ɛ_r_/t*, where *ɛ_r_* is the dielectric constant of triboelectric material [38]. The result of this work showed the opposite trend, which could be explained by the fact that the increasing CNF content suppressed the flexibility of the composite films, due to the decreasing NR polymer fraction. The lower flexibility of the films led to a small change in their thicknesses when they were subjected to a mechanical force, which consequently resulted in the reduction of the TENG output performance. The reduction of film thickness therefore contributed to the drop in TENG outputs in the NR–CNF@1.6% and 2.0%. The optimum CNF of 1.2 wt% was then used to prepare the NR–CNF–AC composites.

The AC, a carbon material with a high specific surface area 1875 m^2^/g (BET), was added to a modified surface morphology of the NR–CNF composite film. Furthermore, AC is a conductive material, and despite its relatively low electrical conductivity, it is able to promote charge capacitance, which is an additional contribution to intensifying the triboelectric charge density of the NR composite films [12,28]. The AC powder at 0.2–0.6 wt% was incorporated into the NR–CNF as well as the NR, for comparison. It was observed that using AC alone could also improve the electrical output of the NR TENG, but there was almost no change in TENG output with AC concentration. This was explained by the fact that the AC particles readily fully covered the film surfaces, and the difference in surface morphologies among the NR–AC@0.2–0.4% was not significant. In the case of the NR–CNF–AC TENG, the improved electrical output was contributed by the electron donor CNF and increased surface area of AC, giving rise to the attained maximum *V_pp_* of 137 V and *I_pp_* of 12.1 µA from the NR–CNF–AC@0.4% TENG. It was noted that the contribution of AC to the NR–CNF–AC composite was higher than for the NR–AC one (AC alone). The synergistic enhancement was ascribed to the interaction between the positive charges of the CNF and the negative surface charges on the AC producing electrical dipoles [39], as well as providing a better dispersion of the AC in the composites. This contributed to the enhancement of the triboelectric charge density and the TENG performance. The summary of *V_pp_* and *I_pp_* of all the fabricated TENGs are displayed in Table 1.

**Figure 7:**
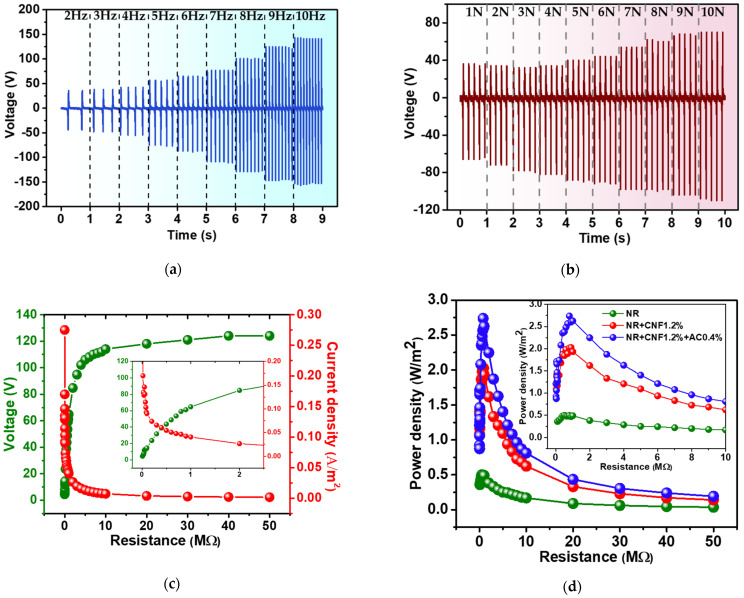
The dependence of (**a**) working frequencies, and (**b**) impact force of the NR–CNF–AC@0.4% TENG; (**c**) output voltage and current measured with the connected load resistances; (**d**) power density derived from (**c**) of the of the NR–CNF–AC@0.4% TENG.

The electrical output of NR–CNF–AC TENGs were found to vary with operating frequency and impact force. The TENG output voltage measured at operation frequencies ranging from 1–10 Hz is presented in Figure 7a. It is seen that output voltage increased with increasing operation frequencies that change from 80 V at 2 Hz to 300 V at 10 Hz. This was ascribed to charge retention or agglomeration, due to short contact cycles at high operation frequency. The dependence of electrical output on impact force was also investigated by measuring output voltage at different impact forces from 1–10 N. It was found that the TENG output voltage increased with increasing applied forces, as displayed in Figure 7b. This was caused by the increasing contact area, due to the deformation of the NR–CNF–AC film under larger impact forces.

The electrical power generated by the NR–CNF–AC@0.4%, NR–CNF and NR TENGs were examined by measuring the output voltage and current when connected to various external load resistances. The power density was then calculated from the formula *P_d_* = *V* × *J,* where *J* is current density (*J = I/area*). Power densities and matched load resistances of NR, NR–CNF and NR–CNF–AC TENGs are listed in Table 2. It was found that NR–CNF–AC TENGs delivered the highest power density of 2.74 W/m^2^, higher than those of the NR–CNF and NR TENGs, which were 2.03 and 0.5 W/m^2,^ respectively. The NR–CNF–AC TENGs showed a superior power output than that of the previously reported TENG, based on NR and CNF materials, as listed in Table 3.

**Figure 8:**
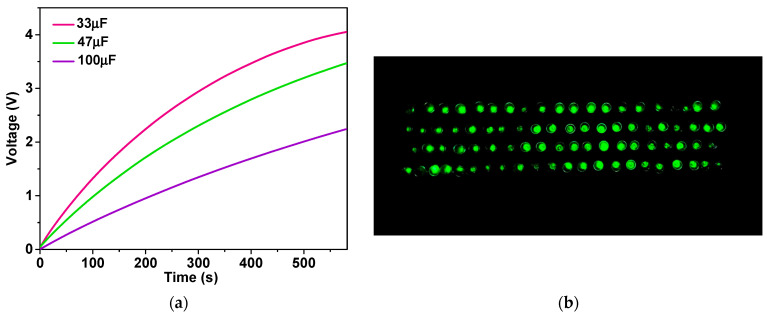
(**a**) Voltage profiles of 33, 47, 100 µF capacitors charged by the fabricated NR–CNF–AC TENG, and (**b**) the application of TENG to power 80 green LEDs.

The power output of the NR–CNF–AC TENG was able to charge commercial capacitors with a bridge rectifier, as demonstrated in the charging profile in Figure 8a. A 100-µF capacitor was charged to 2 V in 500 s, suggesting that the fabricated TENG can be applied as a power source for microelectronic devices. Furthermore, the fabricated TENG was able to instantaneously light up 80 green LEDs as shown in Figure 8b. 

## 4. Conclusions

The electrical output of the NR-based TENG was enhanced by the incorporation of CNF and AC nanoparticles. The NR composite contained CNF at 1.2 wt%, and the AC nanoparticle at 0.4 wt% of NR latex was found to exhibit the highest TENG performance, with the 5–fold improvement of power density. This was attributed to the increased tribopositive polarity by the addition of cellulose and the increased surface area from the presence of the porous nanostructure of the AC. The generated electrical power was demonstrated to charge a wide range of commercial capacitors, which could be applied as a microelectronic power source. The finding of this work has proposed an effective approach to enhance the natural-material-based TENG, which is a green and sustainable power source.

## Figures and Tables

**Table 1:** Electrical output voltage (*V_pp_*) and current (*I_pp_*) of NR, NR–CNF and NR–CNF–AC TENGs.

Specimens	*V_pp_* (V)	*I_pp_* (µA)
NR	57	5.5
NR–CNF@0.8%	107	9.8
NR–CNF@1.2%	113	10.4
NR–CNF@1.6%	106	9.3
NR–CNF@2.0%	108	9.4
NR–AC@0.2%	72	7.0
NR–AC@0.4%	76	7.6
NR–AC@0.6%	74	7.1
NR–CNF–AC@0.2%	131	11.7
NR–CNF–AC@0.4%	137	12.1
NR–CNF–AC@0.6%	127	11.4

**Table 2:** P NR, NR–CNF and NR–CNF–AC TENGs.

TENGs	Power Density (W/m^2^)	Matched Load (MΩ)
NR	0.5	0.5
NR–CNF	2.03	0.9
NR–CNF–AC	2.74	2.8

**Table 3:** Power output performances of NR and CNF-based TENGs.

Materials	Power Density (W/m^2^)	Ref.
NR-TiO_2_	0.237	[10]
CNF paper	0.0106	[40]
CMFs–CNFs	0.0768	[41]
Bacterial Cellulose–ZnO	0.042	[42]
CNF- aminosilane	0.22	[43]
CNF aerogel	2.33	[44]
NR–CNF–AC	2.74	This work

## Data Availability

The data presented in this study are available on request from the corresponding author.

## References

[1] Fan F.-R., Tian Z.-Q., Lin Wang Z. (2012). Flexible triboelectric generator. Nano Energy.

[2] Luo J., Wang Z.L. (2020). Recent progress of triboelectric nanogenerators: From fundamental theory to practical applications. EcoMat.

[3] Shi Q., He T., Lee C. (2019). More than energy harvesting—Combining triboelectric nanogenerator and flexible electronics technology for enabling novel micro-/nano-systems. Nano Energy.

[4] Slabov V., Kopyl S., Soares dos Santos M.P., Kholkin A.L. (2020). Natural and Eco-Friendly Materials for Triboelectric Energy Harvesting. Nano-Micro Lett..

[5] Dudem B., Graham S.A., Dharmasena R.D.I.G., Silva S.R.P., Yu J.S. (2021). Natural silk-composite enabled versatile robust triboelectric nanogenerators for smart applications. Nano Energy.

[6] Luo J., Wang Z., Xu L., Wang A.C., Han K., Jiang T., Lai Q., Bai Y., Tang W., Fan F.R. (2019). Flexible and durable wood-based triboelectric nanogenerators for self-powered sensing in athletic big data analytics. Nat. Commun..

[7] Yang H., Fan F.R., Xi Y., Wu W. (2020). Bio-Derived Natural Materials Based Triboelectric Devices for Self-Powered Ubiquitous Wearable and Implantable Intelligent Devices. Adv. Sustain. Syst..

[8] Ma C., Gao S., Gao X., Wu M., Wang R., Wang Y., Tang Z., Fan F., Wu W., Wan H. (2019). Chitosan biopolymer-derived self-powered triboelectric sensor with optimized performance through molecular surface engineering and data-driven learning. InfoMat.

[9] Zhou J., Wang H., Du C., Zhang D., Lin H., Chen Y., Xiong J. (2022). Cellulose for Sustainable Triboelectric Nanogenerators. Adv. Energy Sustain. Res..

[10] Bunriw W., Harnchana V., Chanthad C., Huynh V.N. (2021). Natural Rubber-TiO2 Nanocomposite Film for Triboelectric Nanogenerator Application. Polymers.

[11] Suphasorn P., Appamato I., Harnchana V., Thongbai P., Chanthad C., Siriwong C., Amornkitbamrung V. (2021). Ag Nanoparticle-Incorporated Natural Rubber for Mechanical Energy Harvesting Application. Molecules.

[12] Chomjun T., Appamato I., Harnchana V., Amornkitbamrung V. (2022). Eco-Friendly Triboelectric Material Based on Natural Rubber and Activated Carbon from Human Hair. Polymers.

[13] Barkakaty B. (2014). Natural Rubber (NR) Biosynthesis: Perspectives from Polymer Chemistry.

[14] Phanthong P., Reubroycharoen P., Hao X., Xu G., Abudula A., Guan G. (2018). Nanocellulose: Extraction and application. Carbon Resour. Convers..

[15] Rajinipriya M., Nagalakshmaiah M., Robert M., Elkoun S. (2018). Importance of Agricultural and Industrial Waste in the Field of Nanocellulose and Recent Industrial Developments of Wood Based Nanocellulose: A Review. ACS Sustain. Chem. Eng..

[16] Zhang R., Dahlström C., Zou H., Jonzon J., Hummelgård M., Örtegren J., Blomquist N., Yang Y., Andersson H., Olsen M. (2020). Cellulose-Based Fully Green Triboelectric Nanogenerators with Output Power Density of 300 W m^−2^. Adv. Mater..

[17] Wang Z.L. (2013). Triboelectric Nanogenerators as New Energy Technology for Self-Powered Systems and as Active Mechanical and Chemical Sensors. ACS Nano.

[18] Yao C., Yin X., Yu Y., Cai Z., Wang X. (2017). Chemically Functionalized Natural Cellulose Materials for Effective Triboelectric Nanogenerator Development. Adv. Funct. Mater..

[19] Niu S., Wang S., Lin L., Liu Y., Zhou Y.S., Hu Y., Wang Z.L. (2013). Theoretical study of contact-mode triboelectric nanogenerators as an effective power source. Energy Environ. Sci..

[20] Kim D., Jeon S.-B., Kim J.Y., Seol M.-L., Kim S.O., Choi Y.-K. (2015). High-performance nanopattern triboelectric generator by block copolymer lithography. Nano Energy.

[21] Zou Y., Xu J., Chen K., Chen J. (2021). Advances in Nanostructures for High-Performance Triboelectric Nanogenerators. Adv. Mater. Technol..

[22] Prada T., Harnchana V., Lakhonchai A., Chingsungnoen A., Poolcharuansin P., Chanlek N., Klamchuen A., Thongbai P., Amornkitbamrung V. (2021). Enhancement of output power density in a modified polytetrafluoroethylene surface using a sequential O2/Ar plasma etching for triboelectric nanogenerator applications. Nano Res..

[23] Wang S., Lin L., Wang Z.L. (2012). Nanoscale Triboelectric-Effect-Enabled Energy Conversion for Sustainably Powering Portable Electronics. Nano Lett..

[24] Kim D., Park S.-J., Jeon S.-B., Seol M.-L., Choi Y.-K. (2016). A Triboelectric Sponge Fabricated from a Cube Sugar Template by 3D Soft Lithography for Superhydrophobicity and Elasticity. Adv. Electron. Mater..

[25] Lee K.Y., Chun J., Lee J.-H., Kim K.N., Kang N.-R., Kim J.-Y., Kim M.H., Shin K.-S., Gupta M.K., Baik J.M. (2014). Hydrophobic Sponge Structure-Based Triboelectric Nanogenerator. Adv. Mater..

[26] Chen J., Guo H., He X., Liu G., Xi Y., Shi H., Hu C. (2016). Enhancing Performance of Triboelectric Nanogenerator by Filling High Dielectric Nanoparticles into Sponge PDMS Film. ACS Appl. Mater. Interfaces.

[27] Mi H.-Y., Jing X., Meador M.A.B., Guo H., Turng L.-S., Gong S. (2018). Triboelectric Nanogenerators Made of Porous Polyamide Nanofiber Mats and Polyimide Aerogel Film: Output Optimization and Performance in Circuits. ACS Appl. Mater. Interfaces.

[28] Harnchana V., Ngoc H.V., He W., Rasheed A., Park H., Amornkitbamrung V., Kang D.J. (2018). Enhanced Power Output of a Triboelectric Nanogenerator using Poly(dimethylsiloxane) Modified with Graphene Oxide and Sodium Dodecyl Sulfate. ACS Appl. Mater. Interfaces.

[29] Bai Z., Xu Y., Li J., Zhu J., Gao C., Zhang Y., Wang J., Guo J. (2020). An Eco-friendly Porous Nanocomposite Fabric-Based Triboelectric Nanogenerator for Efficient Energy Harvesting and Motion Sensing. ACS Appl. Mater. Interfaces.

[30] Mi H.-Y., Jing X., Cai Z., Liu Y., Turng L.-S., Gong S. (2018). Highly porous composite aerogel based triboelectric nanogenerators for high performance energy generation and versatile self-powered sensing. Nanoscale.

[31] Yahya M.A., Mansor M.H., Zolkarnaini W.A.A.W., Rusli N.S., Aminuddin A., Mohamad K., Sabhan F.A.M., Atik A.A.A., Ozair L.N. (2018). A brief review on activated carbon derived from agriculture by-product. AIP Conf. Proc..

[32] Yang P., Shi Y., Li S., Tao X., Liu Z., Wang X., Wang Z.L., Chen X. (2022). Monitoring the Degree of Comfort of Shoes In-Motion Using Triboelectric Pressure Sensors with an Ultrawide Detection Range. ACS Nano.

[33] Gong J., Li J., Xu J., Xiang Z., Mo L. (2017). Research on cellulose nanocrystals produced from cellulose sources with various polymorphs. RSC Adv..

[34] Somseemee O., Saeoui P., Schevenels F.T., Siriwong C. (2022). Enhanced interfacial interaction between modified cellulose nanocrystals and epoxidized natural rubber via ultraviolet irradiation. Sci. Rep..

[35] Chen D., Shao H., Yao W., Huang B. (2013). Fourier Transform Infrared Spectral Analysis of Polyisoprene of a Different Microstructure. Int. J. Polym. Sci..

[36] Zhang Z., Gong W., Bai Z., Wang D., Xu Y., Li Z., Guo J., Turng L.-S. (2019). Oxygen-Rich Polymers as Highly Effective Positive Tribomaterials for Mechanical Energy Harvesting. ACS Nano.

[37] Saqib Q.M., Chougale M.Y., Khan M.U., Shaukat R.A., Kim J., Bhat K.S., Bae J. (2022). Triboelectric nanogenerator based on lignocellulosic waste fruit shell tribopositive material: Comparative analysis. Mater. Today Sustain..

[38] Zhu G., Peng B., Chen J., Jing Q., Lin Wang Z. (2015). Triboelectric nanogenerators as a new energy technology: From fundamentals, devices, to applications. Nano Energy.

[39] Kim M.P., Um D.-S., Shin Y.-E., Ko H. (2021). High-Performance Triboelectric Devices via Dielectric Polarization: A Review. Nanoscale Res. Lett..

[40] Cui P., Parida K., Lin M.-F., Xiong J., Cai G., Lee P.S. (2017). Transparent, Flexible Cellulose Nanofibril–Phosphorene Hybrid Paper as Triboelectric Nanogenerator. Adv. Mater. Interfaces.

[41] He X., Zou H., Geng Z., Wang X., Ding W., Hu F., Zi Y., Xu C., Zhang S.L., Yu H. (2018). A Hierarchically Nanostructured Cellulose Fiber-Based Triboelectric Nanogenerator for Self-Powered Healthcare Products. Adv. Funct. Mater..

[42] Jakmuangpak S., Prada T., Mongkolthanaruk W., Harnchana V., Pinitsoontorn S. (2020). Engineering Bacterial Cellulose Films by Nanocomposite Approach and Surface Modification for Biocompatible Triboelectric Nanogenerator. ACS Appl. Electron. Mater..

[43] Nie S., Cai C., Lin X., Zhang C., Lu Y., Mo J., Wang S. (2020). Chemically Functionalized Cellulose Nanofibrils for Improving Triboelectric Charge Density of a Triboelectric Nanogenerator. ACS Sustain. Chem. Eng..

[44] Zheng Q., Fang L., Guo H., Yang K., Cai Z., Meador M.A.B., Gong S. (2018). Highly Porous Polymer Aerogel Film-Based Triboelectric Nanogenerators. Adv. Funct. Mater..

